# Dissecting the importance and origin of circulating myokines in gastric cancer cachexia

**DOI:** 10.3389/fendo.2024.1437197

**Published:** 2024-10-01

**Authors:** Marek Sierzega, Anna Drabik, Marek Sanak, Robert Chrzan, Piotr Richter

**Affiliations:** ^1^ First Department of Surgery, Jagiellonian University Medical College, Krakow, Poland; ^2^ Faculty of Materials Science and Ceramics, AGH University of Science and Technology, Krakow, Poland; ^3^ Second Department of Internal Medicine, Jagiellonian University Medical College, Kraków, Poland; ^4^ Department of Radiology, Jagiellonian University Medical College, Krakow, Poland

**Keywords:** gastric cancer, cachexia, myokines, interleukins, fatty acid-binding protein 3, follistatin-like 1 protein

## Abstract

**Background:**

Some experimental data suggest that myokines may play an important role in developing cancer-associated cachexia (CAC), but their relevance in humans remains poorly explored. In our study, we tested the hypothesis that circulating myokines are associated with the pathogenesis of CAC in a model population of gastric cancer.

**Methods:**

A group of 171 treatment naïve patients with adenocarcinoma of the stomach were prospectively examined. Cachexia was defined as weight loss >5% or weight loss >2% with either BMI <20 kg/m2 or sarcopenia. A panel of 19 myokines was measured in portal and peripheral blood as well as tumour tissue and surrounding gastric mucosa. Moreover, a serum proteomic signature of cachexia was identified by a label-free quantitative proteomics with a nano LC-MS/MS system and stored in a ProteomeXchange database (PXD049334).

**Results:**

One hundred (58%) patients were diagnosed with CAC. The concentrations of fatty acid-binding protein 3 (FABP3), follistatin-like 1 protein (FSTL−1), interleukin 6 (IL 6), and interleukin 8 (IL 8) were significantly higher in the peripheral blood of cachectic subjects, while leptin levels were lower. Of all the evaluated myokines, tumour tissues showed higher expression levels only for IL-15 and myostatin. However, the analysis of paired samples failed to demonstrate a decreasing concentration gradient between the portal and peripheral blood for any of the myokines, evidencing against their release by the primary tumour. Proteomic analysis identified 28 proteins upregulated and 24 downregulated in the peripheral blood of patients with cachexia. Differentially expressed proteins and 5 myokines with increased serum levels generated a significant protein-protein interaction network.

**Conclusions:**

Our study provides clinical evidence that some myokines are involved in the pathogenesis of cachexia and are well integrated into the regulatory network of circulating blood proteins identified among cachectic patients with gastric cancer.

## Introduction

Cancer-associated cachexia (CAC) is a metabolic wasting syndrome associated with an involuntary progressive loss of skeletal muscle and fat tissue (1). The resulting multifaceted functional impairment is responsible for reduced compliance and efficacy of anticancer treatment, increased treatment-related toxicity, and higher patient mortality (2). Clinical trials utilizing standard regimens of nutritional support showed only moderate efficacy, emphasizing the urgent need for new pharmacological interventions to reverse metabolic disturbances associated with CAC (3–5). Therefore, the search for clinically relevant molecular pathways responsible for the pathogenesis of cachexia is still required.

There is a common consensus that some circulating factors orchestrating the crosstalk between the primary tumor and the involved organs play an important role in the pathogenesis of CAC (6). Since the loss of skeletal muscle is a hallmark of cachexia, much attention has been paid to a rapidly expanding group of signaling molecules called myokines (7). Members of this heterogenous family were shown to act in autocrine, paracrine, or endocrine manners affecting the muscle and distant organs, including adipose tissue, brain, bone, or liver. Although these interactions were shown to contribute to impaired muscle mass and functional changes typical for CAC, some important questions are still to be answered (8, 9). It remains unclear whether there exists a common myokine-dependent mechanism of cachexia. In fact, some previous studies already emphasized marked diversity not only between cancer and non-cancer cachexia, but also between individual cancer types and the prevalence of CAC, possibly related to expression of pro-cachectic mediators (10, 11). Moreover, there have been no satisfactory explanations demonstrating the origins of pro-cachectic factors circulating in the blood. Finally, many previous observations were made solely from experimental data without proper validation in clinical settings.

In this prospective study, we sought to explore the importance of selected myokines in a homogenous population of patients with gastric cancer characterized by high rates of CAC (1). Myokine levels in peripheral blood were compared between patients with and without cachexia to identify mediators potentially involved in the pathogenesis of CAC. Moreover, we compared myokine concentrations in paired samples of portal and peripheral blood, as well as cancer tissue and surrounding healthy mucosa, to verify whether they are released by the primary tumor. Finally, a label-free quantitative proteomics was applied to explore other potential circulating mediators of CAC and to analyze their interactions with myokines.

## Methods

### Patients and treatments

Treatment naïve patients with histologically proven adenocarcinoma of the stomach diagnosed between January 2015 and December 2019 were prospectively examined. The extent of surgery, definitions for lymph node dissection, and tumor staging were adapted to the recent guidelines (12, 13). All data was collected prospectively and recorded in a dedicated database.

### Cachexia and evaluation of nutritional status

Cachexia was defined using international consensus criteria as weight loss >5% over past 6 months, or weight loss > 2% in individuals with body-mass index (BMI) <20 kg/m^2^ or reduced skeletal muscle mass (sarcopenia) (14). Nutritional status was evaluated using clinical (body mass index [BMI], weight loss) and laboratory indices (albumin, C-reactive protein (CRP), lymphocyte counts), as well as composite scores (Nutrition Risk Screening-2002 [NRS-2002], Prognostic Nutrition Index [PNI]). Muscle mass was evaluated using the lumbar skeletal muscle index (SMI) using CT scans as previously described (15).

### Sample processing

Blood samples were collected before starting any tumor-oriented treatment. Peripheral venous blood (5 ml) was collected into sterile BD Vacutainer^®^ tubes under fasting conditions. In a subgroup of consecutive 24 patients selected for proteomic analysis, another 5 ml was drawn intraoperatively from the portal circulation. For paired sample analyses, both blood samples were collected on the same day. After being allowed to clot at room temperature for 60 minutes, the samples were centrifuged at 2000×g for 10 min at 4°C. The serum was removed and stored at −80°C until analysis. Moreover, pairs of primary tumors and corresponding normal gastric mucosa were sampled from patients’ surgical specimens immediately after resection. The fresh specimens were snap frozen in liquid nitrogen and stored at −80°C until processing. Some portions of the collected specimens were used for routine histopathology to verify adequate cellularity of samples corresponding to tumor tissue and normal gastric mucosa.

### Myokine assays

A set of 19 myokines most probably related to cachexia was selected based on previous experimental and clinical studies (8, 9, 16). Serum myokine levels were evaluated using a multiplex immunofluorescent assay platform (Luminex MAGPIX, Merck KGaA, Germany). Human Myokine Magnetic Bead Panel (HMYOMAG-56K, Merck KGaA, Germany) and Human Circulating Cancer Biomarker Magnetic Bead Panel 1 (HCCBP1MAG-58K, Merck KGaA, Germany) were used following the manufacturer’s instructions. Human Parathyroid Hormone Related Protein ELISA Kit (orb406495, Biorbyt Ltd., UK) was used for PTHrP assays.

### Proteomic and bioinformatic analysis

The complete analytical procedures were carried out as detailed in **Supplementary Methods**. Briefly, after selective immunodepletion of albumin and immunoglobulins with the Multiple Affinity Removal System (MARS), serum samples were prefractionated with Pierce™ C18 spin columns. Tissue samples were processed after homogenization. Protein fractions were subsequently separated with a Proxeon nanoscale liquid chromatography system (Bruker Daltonics) and identified using an amaZon ETD mass spectrometer (Bruker Daltonics) (nano LC-MS/MS). For label-free quantitative proteomic analysis, three independent MS runs were completed for each sample and the relative differences in protein levels were determined by the ProfileAnalysis package (Bruker-Daltonics) according to the manufacturers’ recommendations. The acquired spectra were identified using the Mascot algorithm against the Swiss-Prot/TrEMBL sequence database. The mass spectrometry proteomics data have been deposited to the ProteomeXchange Consortium via the PRIDE partner repository with the dataset identifier PXD049334 (17). The Panther classification system was used to identify gene ontology terms (pantherdb.org) (18). Potential associations between proteins related to cachexia identified by myokine assays and proteomic analysis were assessed by the STRING EMBL software (version 11.5, https://string-db.org/) (19).

### Statistical analysis

The differences in proportions between groups were evaluated using the chi-square test, and the Mann–Whitney U test was used to evaluate differences in quantitative variables A *t*-test model was used to evaluate the fold change in the bucket tables generated based on the ProfileAnalysis. Principal Component Analysis (PCA) with a scaling algorithm was conducted for data overview. The significance level (*P*) <0.05 in a two-tailed test was considered statistically significant. All statistical analyses were performed using the IBM^®^ SPSS^®^ Statistics 28 software package (IBM Corporation, NY) and RStudio (Integrated Development Environment for R) version 2021.9.2.382.

## Results

### Cachexia and clinical parameters

A total of 171 patients with gastric cancer diagnosed between January 2015 and December 2019 were recruited to participate in the study. Cachexia was diagnosed in 100 of 171 (58%) subjects. The comparison of demographic and clinical characteristics between patients with and without cachexia is summarized in **Table 1**. Generally, patients with cachexia were older and had clinical and laboratory findings typical for malnutrition. Gastric cancers among cachectic patients were larger and had more advanced stages. The absence of cachexia was associated with a significantly higher proportions of resectable (95% *vs* 83%, *P*=0.019) and curatively resected (95% *vs* 69%, *P*=0.001) disease.

**Table 1:** Comparison of clinicopathological features between patients with and without cachexia.

Characteristics	No cachexia (n=71)	Cachexia (n=100)	P
Age	63 (57–72)	64 (60–73)	0.143
Males	46 (65%)	55 (55%)	0.200
Body mass index (BMI)	25.9 (24.1–27.9)	22.9 (20.5–26.1)	0.001
Serum albumin (g/L)	43.0 (41.0–45.8)	41.0 (38.5–44.0)	0.030
Serum protein (g/L)	69.5 (66.0–73.0)	68.0 (64.5–70.3)	0.018
Total cholesterol (mmol/L)	4.21 (4.03–4.49)	3.92 (3.74–4.19)	0.030
Haemoglobin (g/L)	13.6 (11.5–14.3)	12.6 (10.8–13.7)	0.008
Lymphocytes (per mm^3^)	1.97 (1.40–2.20)	1.62 (1.10–1.90)	0.026
CRP (mg/L)	2 (1–3)	3 (1–9)	0.289
Prognostic Nutrition Index (PNI)	44.1 (42.0–46.0)	41.6 (39.4–44.8)	0.019
Nutritional risk screening 2002 (NRS2002) 1 2 3 4	48 (68%) 22 (31%) 1 (1.4%) 0	46 (46%) 29 (29%) 14 (14%) 11 (11%)	0.001
Preoperative weight loss (%)	0 (0–3)	13 (9–18)	0.001
Diabetes mellitus	12 (18%)	15 (19%)	0.891
L3 lumbar skeletal muscle index (SMI) females males	46 (40–52) 61 (53–64)	38 (34–44) 49 (37–52)	0.046 0.013
Muscle density (HU)	31 (27–40)	33 (27–36)	0.993
Charlson Comorbidity Index 1 2 3 or more	40 (59%) 18 (26%) 10 (15%)	39 (48%) 17 (21%) 25 (31%)	0.068
ASA Physical Status, 3–4	12 (18%)	12 (15%)	0.639
Tumour size (mm)	45 (30–62)	60 (40–90)	0.003
Tumour location upper third middle third distal third whole stomach	17 (24%) 28 (39%) 22 (31%) 4 (6%)	21 (21%) 37 (37%) 21 (21%) 17 (17%)	0.162
Lauren classification, diffuse	47 (69%)	60 (74%)	0.503
Tumour grade, moderate or poor	67 (99%)	78 (96%)	0.626
Tumour stage (AJCC 2010) I II III IV	14 (20%) 22 (31%) 29 (41%) 6 (8%)	7 (7%) 12 (12%) 46 (46%) 35 (35%)	0.001
Resection	68 (96%)	82 (82%)	0.007
Curative resection (R0)	60 (88%)	61 (59%)	0.001

Data are expressed as median (interquartile range) or number (%); the Mann–Whitney U test was used to evaluate differences in quantitative variables and the differences in proportions were evaluated using the chi-square test.

CRP, C-reactive protein; ASA, American Society of Anesthesiologists; AJCC, American Joint Committee on Cancer.

### Peripheral blood myokines

**Figure 1:**
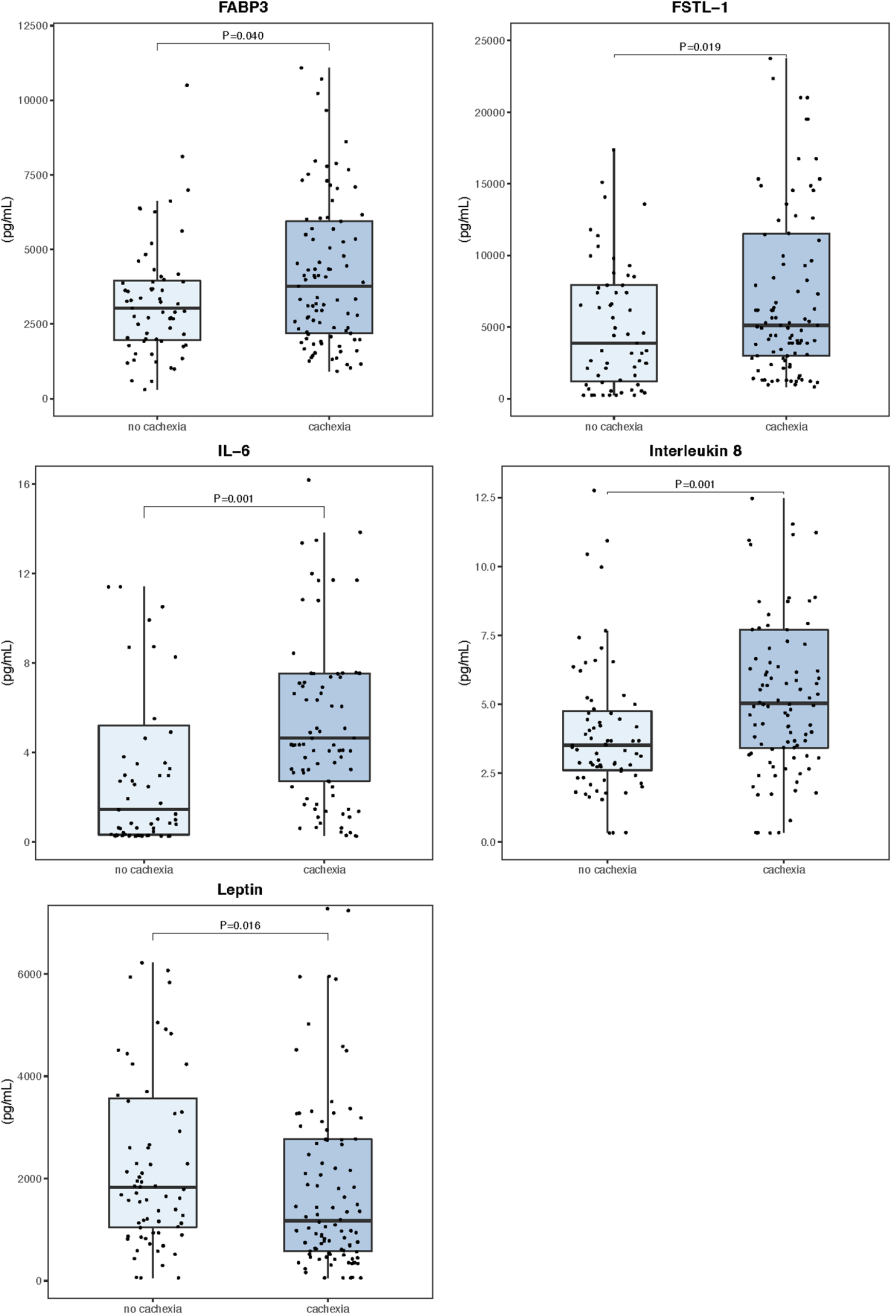
Serum myokine concentrations in the peripheral blood (n=171). Data are expressed as median (center line) and interquartile range (box). Mann–Whitney U test was used to compare median values between patients with and without cachexia.

The peripheral levels of circulating myokines in patients with and without cachexia are summarized in **Table 2** and sex-related variability is presented in **Supplementary Table S1**. Patients with cachexia had significantly higher levels of fatty acid-binding protein 3 (FABP3), follistatin-like 1 protein (FSTL−1), interleukin 6 (IL 6), and interleukin 8 (IL 8), while leptin concentrations in the peripheral blood were significantly lower (**Figure 1**). Individual myokines were poor predictors of cachexia with AUC for ROC curves not exceeding 0.690 (**Supplementary Table S2**, **Supplementary Figure S1**).

**Table 2:** Peripheral blood levels of myokines.

Concentration (pg/mL)	No cachexia (n=71)	Cachexia (n=100)	P
Apelin	12 (0, 22)	12 (0, 20)	0.782
BDNF	3161 (2161, 4161)	3437 (2853, 4382)	0.166
Erythropoeitin	648 (56, 811)	124 (14, 936)	0.417
FABP3	3032 (1958, 3948)	3768 (2186, 5939)	0.040
FGF2	40 (26, 61)	40 (24, 50)	0.580
FGF21	8 (5, 141)	13 (5, 113)	0.855
Fractalkine	54 (13, 134)	95 (13, 189)	0.197
FSTL-1	3872 (1204, 7914)	5114 (2988, 11502)	0.019
Interleukin 6	1 (0, 5)	5 (3, 8)	0.001
Interleukin 8	3.5 (2.6, 4.8)	5.0 (3.4, 7.7)	0.001
Interleukin 15	0.92 (0.92, 1.51)	1.14 (0.92, 2.85)	0.629
Irisin	75 (0, 165)	85 (0, 220)	0.973
LIF	2.4 (1.8, 2.9)	2.8 (1.6, 3.8)	0.862
Leptin	1834 (1049, 3570)	1174 (576, 2766)	0.016
Myostatin	126 (116, 289)	178 (116, 537)	0.983
Oncostatin M	15 (7, 25)	14 (9, 24)	0.958
Osteocrin	13 (13, 18)	13 (3, 23)	0.624
Osteonectin	156 (120, 189)	152 (117, 191)	0.909
PTHrP	1871 (1189, 3793)	1965 (975, 3535)	0.539

Data are expressed as median (interquartile range). Mann–Whitney U test was used to compare groups. BDNF, Brain-Derived Neurotrophic Factor; FABP3, Fatty Acid-Binding Protein 3; FSTL-1, Follistatin-Like 1 Protein; FGF21, Fibroblast Growth Factor 21; FGF2, Fibroblast Growth Factor 2; LIF, Leukemia Inhibitory Factor; PTHrP, Parathyroid Hormone-Related Protein.

**Figure 2:**
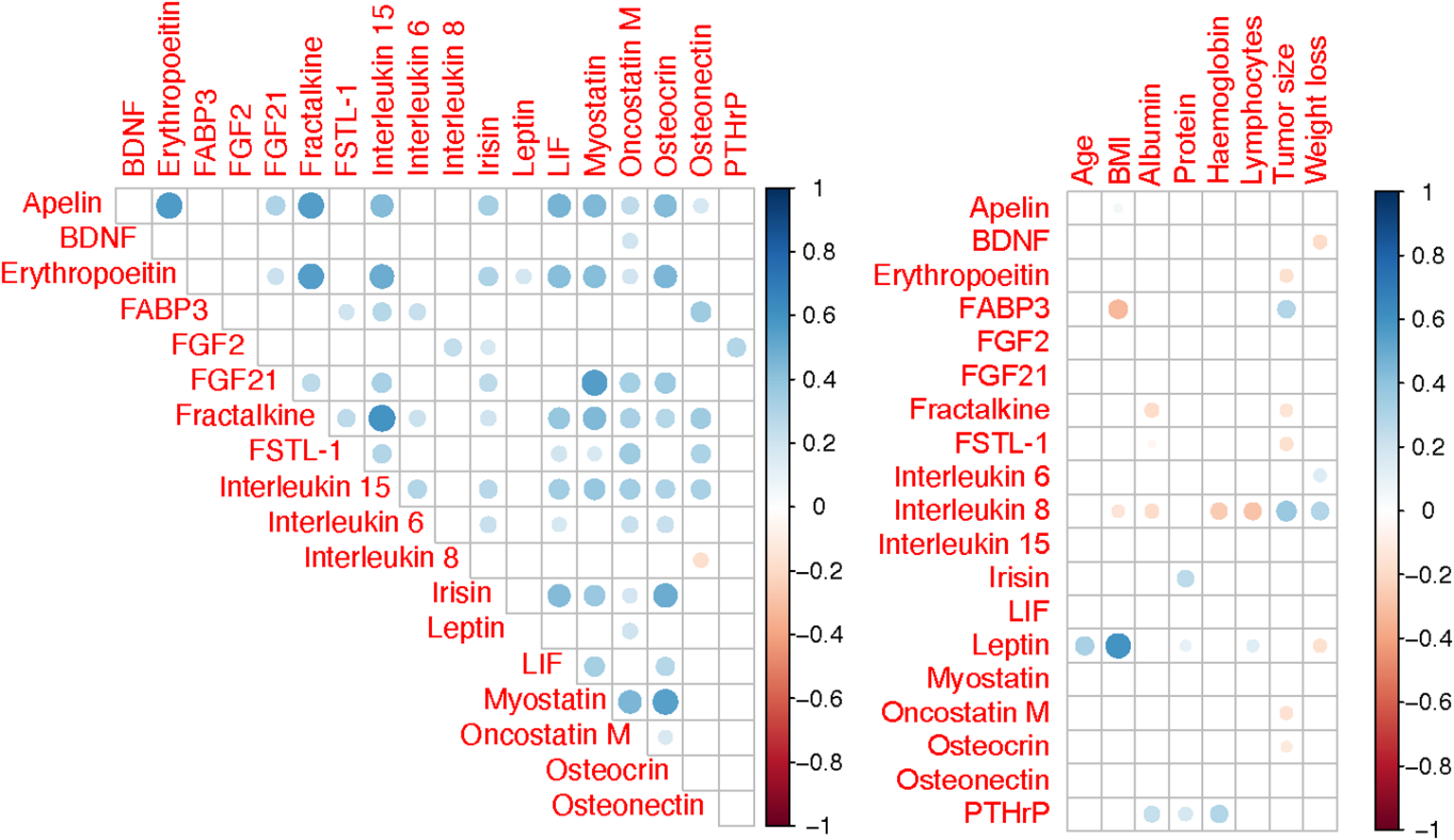
Spearman correlation matrix for variables. Only correlations with *P <*0.05 are shown and correlation strength is indicated by colour intensity.

**Figure 2** shows results of the correlation analysis between individual myokines as well as myokines and clinical variables. Of all tested myokines, only leptin showed significant age- and sex-dependent variability. Overall blood levels of leptin were higher in females (2229 vs 1126 pg/mL, *P*=0.017) and were positively correlated with age (R=0.22, *P*=0.012). There was a weak negative association of patients’ BMI with FABP3 (R= –0.20, *P*=0.011) and IL 8 (R= –0.18, *P*=0.017), while leptin levels showed a moderate positive correlation (R= 0.59, *P*=0.001) (**Supplementary Figure S2**). Furthermore, leptin was negatively correlated with preoperative percentage weight loss (R= –0.23, *P*=0.003), but IL 6 (R= 0.27, *P*=0.001) and IL 8 (R= 0.32, *P*=0.001) showed positive correlations (**Supplementary Figure S3**). FSTL−1, FABP3 and IL 6 were not associated with tumor stage (**Supplementary Figure S4**). However, serum levels of interleukin 8 were significantly lower among patients with stage I/II disease compared to stage III and IV. An opposite relationship was found for leptin, where median levels for stage IV disease (776 pg/mL) were higher than either I/II (1834 pg/mL, *P*<0.001) or III (1637 pg/mL, *P*=0.006).

### Portal blood and tissue myokines

**Figure 3:**
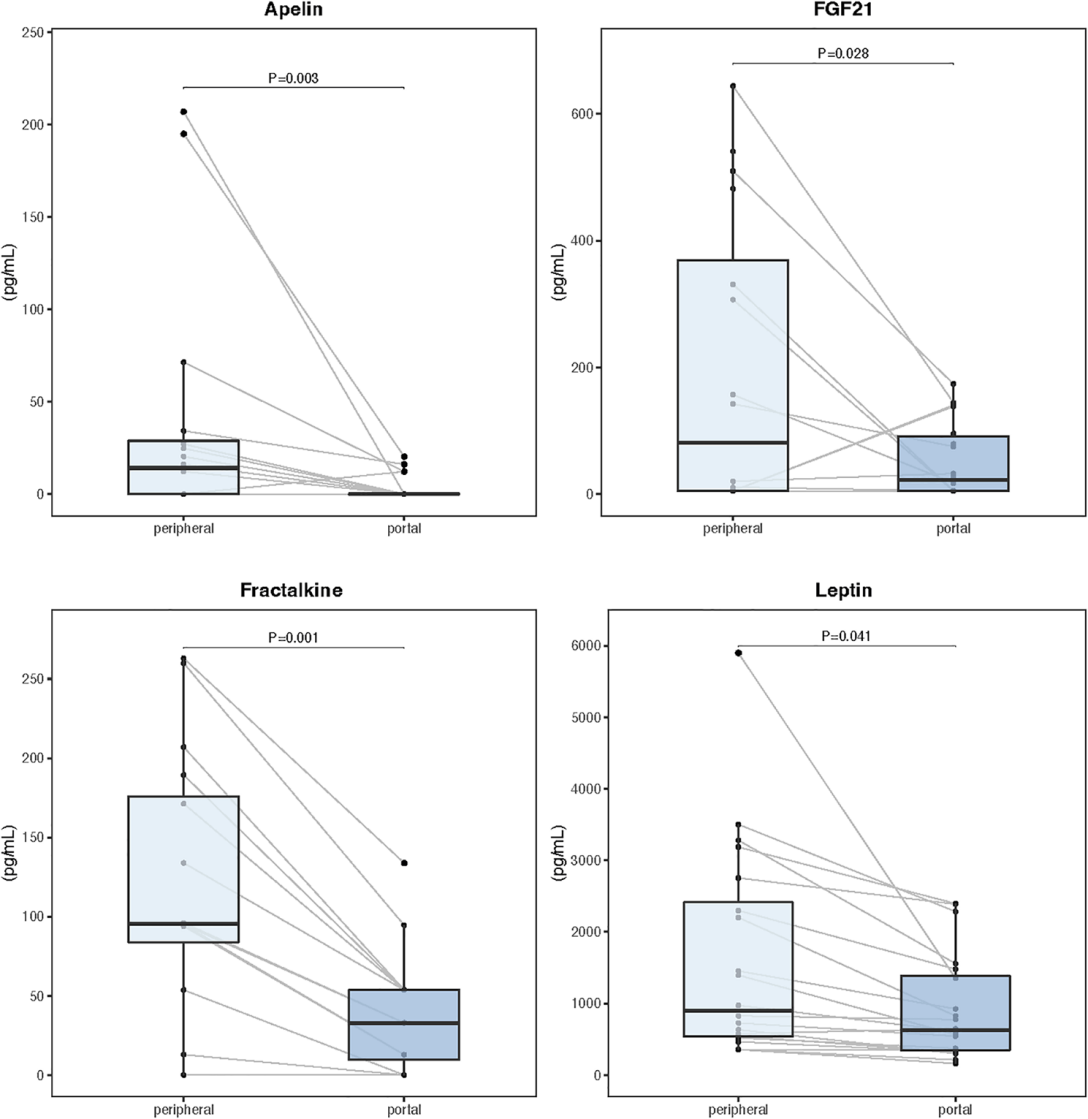
Myokine concentrations in paired samples of portal and peripheral blood (n=24). Data are expressed as median (center line) and interquartile range (box). Wilcoxon signed-rank test was used to compare paired samples.

Serum and tissue samples from 24 patients, including 16 diagnosed with cachexia, were selected for comparative myokine analysis and label-free quantitative proteomics. There were no significant differences in demographic and clinical characteristics between this subgroup and the overall population. Analysis of paired portal and peripheral blood samples failed to identify any myokine with elevated levels in the portal circulation (**Supplementary Table S3**). However, portal concentrations of apelin, fibroblast growth factor 21 (FGF21), fractalkine, and leptin were significantly reduced compared to the peripheral blood (**Figure 3**). **Supplementary Figure S5** shows Spearman correlation matrix for portal and peripheral blood levels of myokines.

Relative myokine expression between the primary tumor and adjacent healthy gastric mucosa was evaluated by LC-MS/MS. No signal for erythropoeitin, fractalkine, leukemia inhibitory factor (LIF), osteocrin, and osteonectin could be detected in the samples. Of all the remaining myokines, tumor tissues showed higher expression only for IL-15 and myostatin with relative abundance ratios of 2.4 and 3.1, respectively.

### Proteomic profiling of peripheral blood

**Figure 4:**
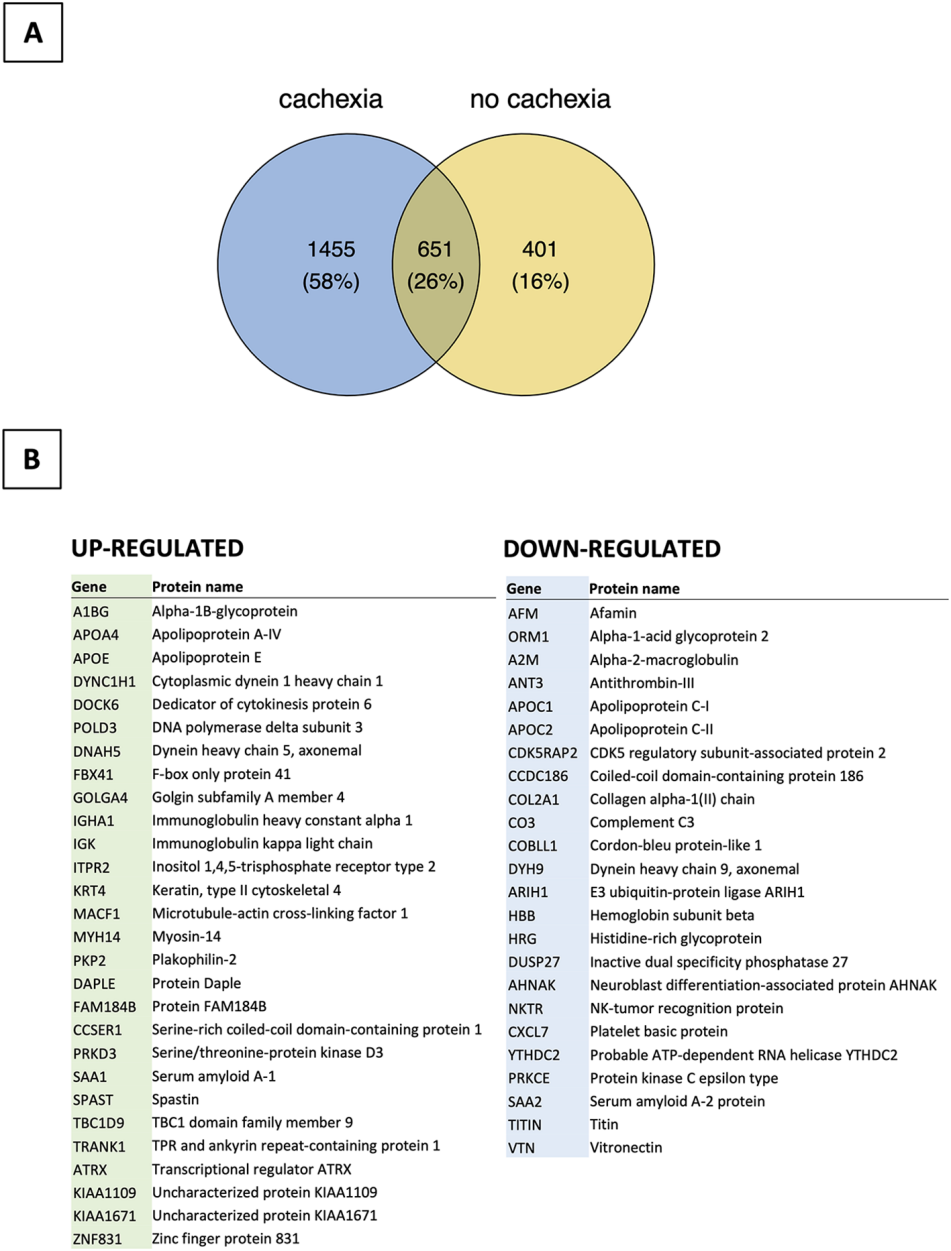
Bioinformatics analysis of proteomic data (n=24). **(A)** Distribution of proteins detected in patients with and without cachexia. **(B)** List of up- and down-regulated proteins among cachectic patients.

**Figure 5:**
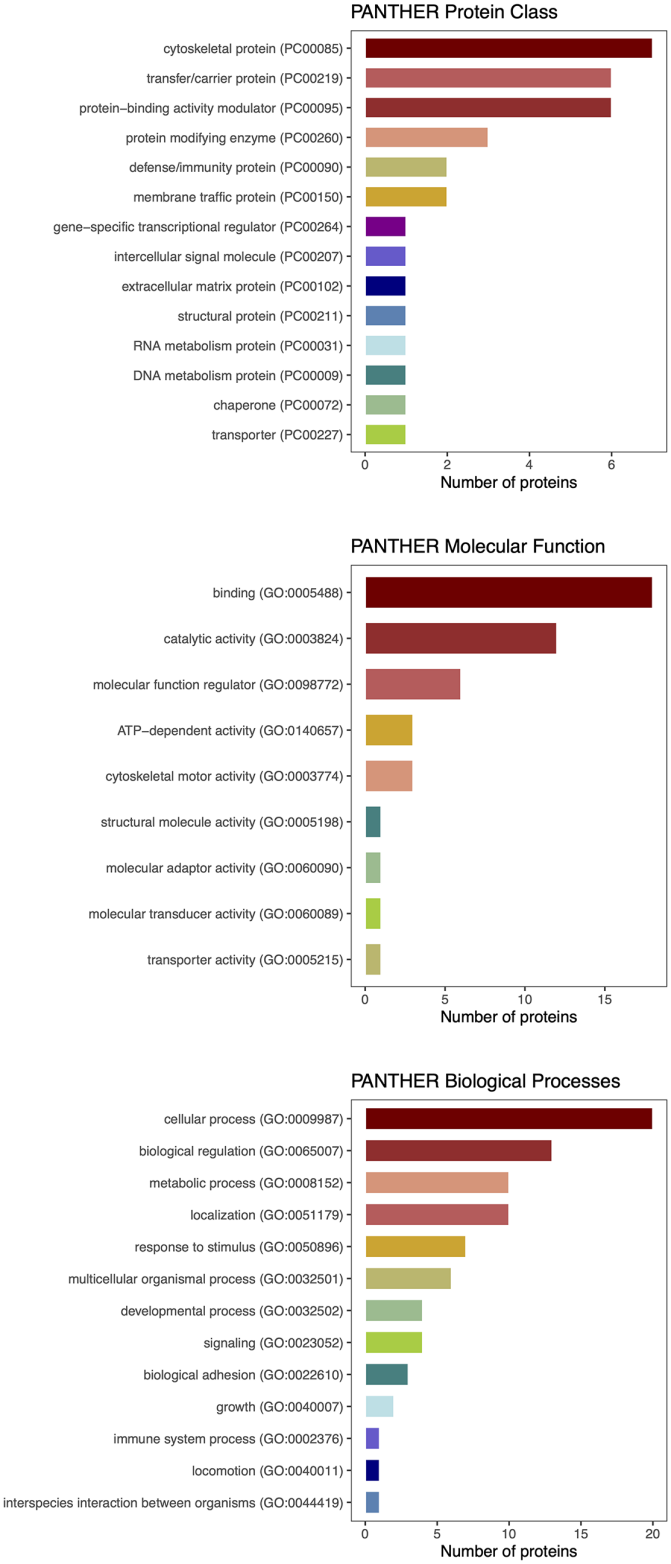
PANTHER classification of differentially expressed proteins detected by proteomic analysis.

**Figure 6:**
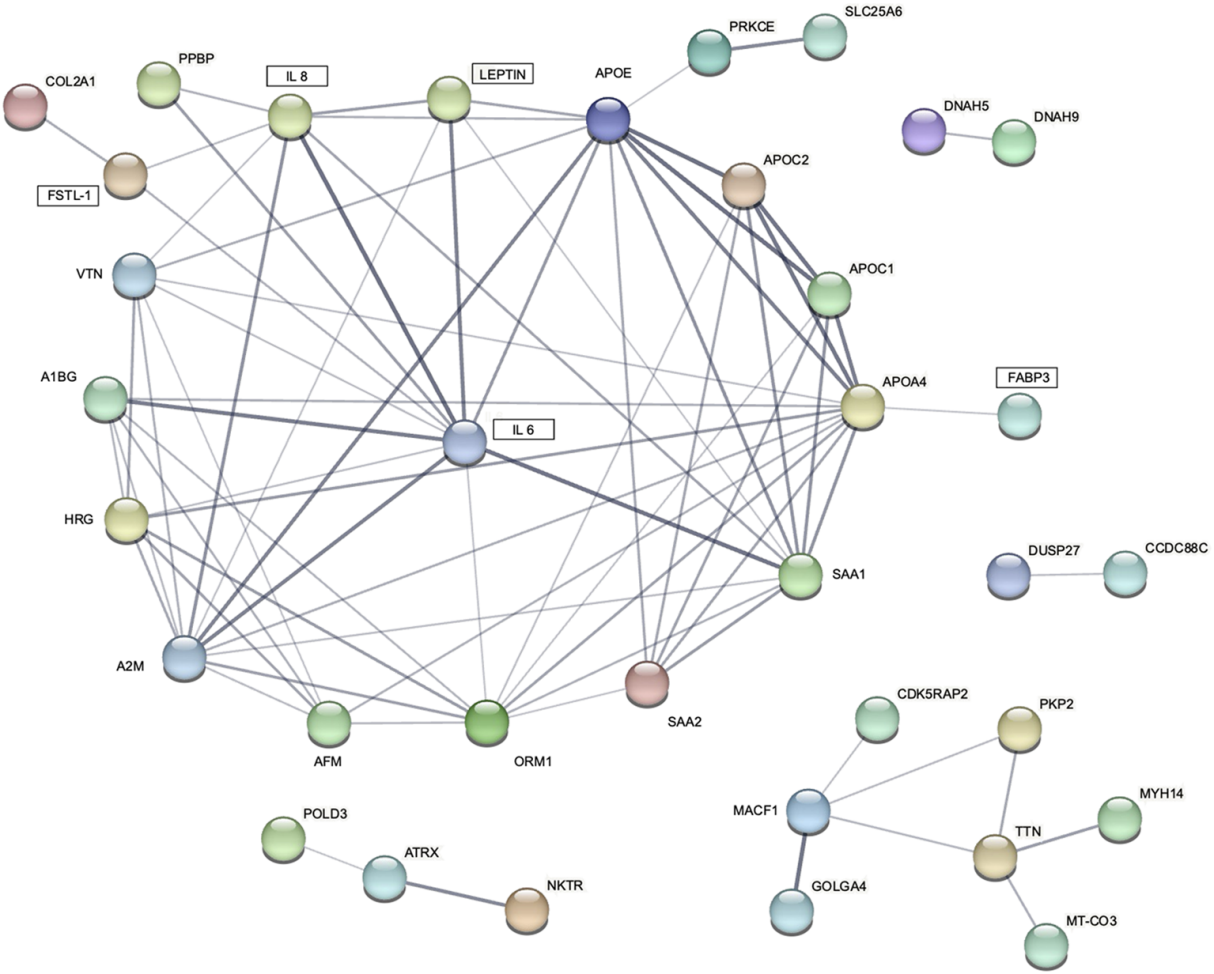
STRING protein-protein interaction (PPI) network for 5 differentially expressed blood myokines (rectangles) and proteins identified by proteomic analysis. Disconnected nodes are hidden. Line thickness indicates the strength of data support.

The proteomic analysis identified 2507 unique proteins in all serum samples, including 1455 found only in patients with cachexia (**Supplementary Tables S4**, **S5**). There were 28 proteins upregulated and 24 downregulated among cachectic patients (**Figures 4**, **5**). The protein-protein interaction (PPI) network for all 52 differentially expressed proteins and 5 myokines with increased serum levels generated 55 nodes and 77 edges with an average node degree of 2.8 and the PPI enrichment *P* value of 10^-16^ (**Figure 6**).

## Discussion

This study carried out a comprehensive profiling of circulating myokines in a population of Western patients with gastric cancer. Five myokines were found to be potentially associated with cancer cachexia, but none of them were released by the primary tumor. However, we demonstrated that all these myokines were well integrated into the interaction network of circulating proteins identified among cachectic patients by proteomic profiling.

Data from animal and *in silico* models sufficiently demonstrated a complex network of interactions between several active mediators potentially associated with cachexia, including cytokines, myokines, adipokines, and tumor factors (20–24). However, there is still a translational gap between human and animal studies, which prevents a simple extrapolation of experimental results. This is particularly relevant for myokines, where most clinical studies measured only a limited number of mediators in relatively small populations recruiting patients with diverse cancers (25, 26).

Myokines are a heterogeneous group that includes more than 600 different signaling molecules produced and released by skeletal muscles (7). Their biological function is to control myocyte activity through an autocrine loop, as well as to provide communication with other tissues and organs (paracrine and endocrine effects). Interleukin 6 (IL-6) derived from skeletal muscles has been identified as the first myokine secreted into the systemic circulation (27) Subsequent clinical and experimental studies have demonstrated its pleiotropic activity and its multifaceted role in cancer cachexia (7, 28). The Janus-faced behavior of IL-6 is related to the fact that circulating cytokine promotes chronic inflammation leading to catabolic changes and skeletal muscle atrophy, while released from myocytes improves muscle metabolism by anti-inflammatory effects. Interleukin-8 (IL-8), a member of the chemokines family, is an inflammatory mediator that exerts chemoattractant activity for lymphocytes and neutrophils, as well as promotes angiogenesis (29). IL-8 was also found in skeletal muscles after exercise, but its physiological function as a myokine remains largely unknown. Fatty acid binding protein 3 (FABP3) is mainly responsible for intracellular transport of lipids, and high expression levels are found in cardiac and skeletal muscles (30). Various types of muscle injury are associated with an increase in circulating FABP3, and some studies suggested that this myokine may have a protective effect against oxidative stress (31). Follistatin-like protein 1 (FSTL-1) is found in most tissues, including cardiomyocytes and skeletal muscles (32). FSTL1 is related to the activation of angiogenic factors, possibly related to vascularization necessary for muscle fiber regeneration (33). Leptin, initially treated as a classic adipokine, was subsequently identified also in skeletal muscles along with abundant expression of its receptors (34). In addition to its metabolic effects, which involve anti-lipogenic activity and improved glucose disposal in skeletal muscles, myokine increases proliferation playing the role of a muscle growth factor (35).

The current study enrolled a homogeneous population of patients with gastric cancer characterized by a high prevalence of cachexia to evaluate the role of myokines in the pathogenesis of CAC. Based on data from previous observations, mostly conducted in experimental settings, 19 myokines possibly associated with cachexia were selected (1, 6–9, 36). We found that peripheral serum levels of FABP3, FSTL−1, interleukin 6, and interleukin 8 were significantly higher in cachectic patients, while leptin levels were decreased. Several previous studies reported similar observations for IL 6, IL 8, and leptin (25, 26). However, very little human data is available for the other two myokines identified in this study, i.e., FABP3 and FSTL−1. Recently, de Castro et al. evaluated plasma and tumor levels of selected myokines (IL 6, IL 8, IL 15, FABP3, FSTL−1, BDNF, irisin, and myostatin) in 94 patients with gastric or colorectal cancers (37). Of the eight candidate biomarkers, higher blood levels were found among cachectic subjects only for IL 6, IL 8, and FABP3. No further clinical data could be identified linking FABP3 or follistatin-like protein 1 (FSTL−1) with cachexia (38–40).

One of the unresolved issues related to the mechanisms of CAC is the source of circulating mediators. For gastrointestinal malignancies, any tumor-derived signalling molecule released into the portal blood must pass through the liver before reaching the systemic circulation. Studies evaluating hepatic clearance of IL 6 and IL 8 among subjects with normal liver function suggested either decreased (41, 42) or unchanged (43–45) concentrations in the peripheral blood compared to the portal compartment. Therefore, cytokines measured in routine blood samples do not necessarily represent the cytokine profile released by gastric cancers and no appropriate studies were carried out for most myokines. To verify the hypothesis that the primary tumor was responsible for releasing factors inducing cachexia, we compared myokine concentrations in paired samples of portal and peripheral blood, as well as cancer tissue and surrounding healthy mucosa. Relative tissue myokine expression evaluated by the LC-MS/MS method showed increased tumor levels only for IL-15 and myostatin, arguing against the possibility of increased production of cachexia related mediators within the tumor. Moreover, the analysis of paired samples from tumor-draining veins and the peripheral circulation failed to demonstrate a decreasing concentration gradient between the portal and peripheral blood for any of the evaluated myokines, including those associated with cachexia (FABP3, FSTL−1, IL 6, IL 8, leptin). In fact, leptin levels were significantly higher in the peripheral circulation. Altogether, these observations clearly demonstrated that the origin of circulating myokines associated with CAC was different from the primary tumor.

Given the paucity of human data, mechanisms involving circulating myokines in CAC are poorly understood. Therefore, our objective was to examine the network of cachexia-related mediators using a label-free quantitative proteomic approach. Analysis of serum samples from patients with and without CAC applying the proteomic workflow based on a nano LC-MS/MS system identified 52 differentially expressed proteins. We interrogated molecular pathways of these proteins and five differentially expressed myokines by the protein–protein interaction (PPI) network analysis using the STRING database. The *P* value of the PPI enrichment model shoved a highly significant network of interactions. There were 60 gene ontology biological processes related to the identified myokines, including 23 for leptin and FABP3, 21 for IL 6, and 8 for IL 8.

Very few studies have used complex proteomic profiling of patients with cachexia, and most of them were focused on muscle samples (46–50). To the best of our knowledge, only one previous attempt has been made to characterize circulating blood proteins in this group of patients. Narasimhan et al. used an aptamer-based platform to screen 1294 plasma proteins from 30 patients with pancreatic ductal adenocarcinoma using weight loss of at least 5% during the prior 6 months as the definition of cachexia (51). They found 67 differentially expressed proteins, including 10 up-regulated and 57 down-regulated. Although no protein overlapped with the current study, leptin correlated with weight loss grade, skeletal muscle index, and total adipose index, while FABP3 correlated with skeletal muscle density. Moreover, IL 6 was suggested as one of the possible upstream regulators for molecules involved in the pathogenesis of cachexia.

The results of this study provide important clinical evidence for further research focused on circulating myokines in the pathogenesis of cancer-associated cachexia. However, certain important limitations should be considered. First, we were unable to provide some functional parameters associated with cachexia, including quality of life and muscle strength. Although such information was not necessary to diagnose cachectic patients, it could provide some additional insights. Second, we did not validate the results of proteomic analyses using other methods, such as ELISA. However, the main aim of proteomic profiling in this study was to dissect protein-protein interaction networks of myokines potentially involved in CAC instead of validation of new cachexia-associated biomarkers. Third, we used ‘myokines’ to designate the molecules evaluated in our study. All were selected based on previous reports suggesting their pivotal role in muscle–organ crosstalk potentially related to cachexia. However, by definition, myokines refer to a group of mediators that are produced and released by skeletal muscle cells. All myokines identified in our study as potentially associated with cancer cachexia are normally released by various cells, predominantly within the immune system (IL-6, IL−8, FABP3) and adipose tissue (leptin, FSTL-1). Since there is no analytical method currently available to determine the actual source of circulating mediators, there is still a gap in mechanistic understanding of the members of the myokine family found in blood.

Overall, our study provides clinical evidence that some myokines are involved in the pathogenesis of cachexia and are well integrated into the regulatory network of circulating blood proteins identified among cachectic patients. As none of these myokines was released by the primary tumor, this has important implications for further studies on the pathogenesis of cancer-associated cachexia.

## Data Availability

The datasets presented in this study can be found in online repositories. The names of the repository/repositories and accession number(s) can be found below: https://www.ebi.ac.uk/pride/archive/, PXD049334.

## Supplementary material

Supplementary Methods Detailed description of label-free quantitative proteomics with a nano LC-MS/MS system.
