# Supplementary material for: Dissecting the importance and origin of circulating myokines in gastric cancer cachexia
Source: Front Endocrinol (Lausanne). 2024 Oct 1;15:1437197. doi: 10.3389/fendo.2024.1437197 (PMC11473381; doi:10.3389/fendo.2024.1437197)
Supplement: Supplementary Methods — Detailed description of label-free quantitative proteomics with a nano LC-MS/MS system. [file DataSheet1.docx]

*Sample preparation for MS analysis*

Immunodepletion of albumin and IgG was performed using the Multiple Affinity Removal System (MARS) HSA/IgG spin cartridge (Agilent) according to the manufacturer’s protocol. Serum samples of 50 μL were diluted with Buffer A (Agilent) to 200 μL and filtered through a 0.22 μm spin filter (Costar Spin-X) in Protein LoBind tubes (Eppendorf). After equilibration with 4 mL of Buffer A, the spin cartridge was loaded with 200 μL of the filtered serum and centrifuged for 1.5 min at 100×g. After washing the column twice with 400 μL of buffer A, the flow-through fractions, representing depleted serum, were collected. The bound proteins were released with 2 mL elution of buffer B using the Luer lock adapter and a syringe. The cartridge was re-equilibrated using a syringe with 4 mL of buffer A, and the following sample was immunodepleted.

Prefractionation of proteins from immunodepleted sera was performed separately for each sample using disposable C18 Spin columns (Pierce) and the following fractions were eluted applying 200 μL of (1) 5%ACN 0.5%TFA, (2) 10%ACN 0.5%TFA, (3) 25%ACN 0.5%TFA, (4) 50%ACN 0.5%TFA, and (5) 80%ACN 0.5%TFA after column resin activation with 50%MeOH and equilibration with 5%ACN 0.5%TFA. The obtained fractions were evaporated to dryness in a vacuum centrifuge (Labconco).

Proteins prevalent in fractions form each serum sample were dissolved in 20 μL of 50mM NH_4_HCO_3_ followed by the addition of 5 μL of 50mM 1,4 dithiothreitol DTT (Sigma-Aldrich) and 10 min incubation at 90°C. The reduced proteins were carbamidomethylated by the addition of 5 μL of 100 mM iodoacetamide (IAA) (Sigma-Aldrich) during incubation for 10 min at 90°C. Samples were digested using 5 μL of 0.1 μg/μL trypsin (Promega) in 50mM NH_4_HCO_3_. The digestion was performed for 16 h at 37°C in a thermomixer (Eppendorf). Subsequently, the samples were evaporated to dryness in a vacuum centrifuge. Finally, the resulting peptides were dissolved in 20 μL of 2%ACN 0.1% FA (Sigma-Aldrich).

*Mass spectrometry analysis*

The coupling of nanoLC-MS/MS was used for separation and identification of the obtained protein digests. Protein separation was carried out with a Proxeon nanocapillary chromatography system (Bruker Daltonics) equipped with the PepMap column containing reversed phase material (15 cm long, 75 μm ID, C18, 3 μm particle size), and a precolumn comprising the same packing material but 5 μm particle size. The gradient was formed using 2%ACN 0.1% FA in water (solvent A) and 80%ACN 0.1% FA (solvent B), and it was delivered at a flow rate of 300 nL/min. The system was controlled by Hystar software (Bruker Daltonics). A gradient was produced from 0 to 50% B in 30 min and up to 90% B at 35 min, then kept until 45 min, and again reduced to 0% until 55 min. The chromatographic system was directly coupled to the amaZon ETD mass spectrometer (Bruker Daltonics) operating in a positive-ion mode. For the duration of analysis, the two most intense peaks (threshold above 100,000) in the range 450−1900 m/z were automatically fragmented using CID in data-dependent acquisition mode. Active exclusion parameters include exclusion after two spectra with absolute intensity thresholds over 250,000 and release after 1 min. Charge state parameters include preferable charge state 2+ and 3+; however, other charge states were not excluded. ICC target was selected as 40,000. Accumulation time to achieve this number of ions entering the trap was less than 1 ms. Trap drive was set as 35. Two technical replicates were run, respectively for protein identification.

*Bioinformatic analysis*

The acquired spectra were analyzed using Bruker Data Analysis 4.1 software (DA.xml method script) and were identified using the Mascot 2.4.1 algorithm against the Swiss-Prot/TrEMBL sequence database 2018_03 (557012 sequences; 199714119 residues). Search parameters were set as follows: taxonomy, human; modification, carbamidomethyl (fixed) or methionine dioxidation (variable); up to 1 missed cleavage; peptide charges +1, + 2, and +3; mass tolerance, 0.8 Da for precursor mass and 0.6 Da for fragment mass. Proteins with at least two fragmented unique peptides detected were considered with an additional criterion of an ion score above 40, which is due to the level of false positives p ≤ 0.05. Based on the acquired results fractions 1-4 were tendered for following analyses, while fraction 0 (C18 Spin Cartridge flow-through) and 6 (80%ACN 0,5%TFA) were excluded from further experiments because of the low protein capacity.

For label-free quantitative proteomic analysis three independent MS runs were completed for each sample using identical mass spectrometer parameters as in MS/MS mode, though without fragmentation. A molecular feature script was created using Data Analysis Software (FMF.xml). Profile Analysis package (Bruker-Daltonics) was used for establishing the differences in protein levels between studied patients’ groups according to the manufacturers’ recommendations (Label free quantitation workflow). T-test model was used to calculate the fold change in the bucket tables generated based on the MS analysis results. Both quantitative and qualitative data were transferred to the Protein Scape 3.1.5 Workstation Server (Bruker-Daltonics), after that results of all fractions were combined. Principal Component Analysis PCA was achieved with a scaling algorithm and confidence level 95%.
