# Supplementary material for: Dissecting the importance and origin of circulating myokines in gastric cancer cachexia
Source: Front Endocrinol (Lausanne). 2024 Oct 1;15:1437197. doi: 10.3389/fendo.2024.1437197 (PMC11473381; doi:10.3389/fendo.2024.1437197)
Supplement: Supplementary file 2 [file DataSheet2.docx]

**Table S1.** Sex-related comparison of peripheral blood myokines between cachectic and non-cachectic patients

| **Concentration (pg/mL)** | **Females** | | ***P*** | **Males** | | ***P*** |
| --- | --- | --- | --- | --- | --- | --- |
|  | no cachexia  (N = 25) | cachexia (N = 45) |  | co cachexia (N = 46) | cachexia (N = 55) |  |
| Apelin | 16 (0, 22) | 6 (0, 21) | 0.640 | 12 (0, 22) | 12 (0, 20) | 0.950 |
| BDNF | 3268 (2182, 4483) | 3392 (2819, 4472) | 0.214 | 2978 (2119, 4161) | 3742 (2865, 4236) | 0.173 |
| Erythropoeitin | 612 (0, 721) | 98 (14, 1148) | 0.842 | 648 (56, 1089) | 156 (21, 778) | 0.177 |
| FABP3 | 2805 (1454, 3830) | 4234 (2530, 6214) | 0.024 | 3106 (2157, 5254) | 3264 (2156, 3986) | 0.675 |
| FGF2 | 45 (31, 68) | 40 (16, 50) | 0.201 | 37 (21, 57) | 37 (28, 50) | 0.733 |
| FGF21 | 22 (5, 108) | 11 (5, 128) | 0.903 | 7 (5, 144) | 16 (5, 58) | 0.963 |
| Fractalkine | 54 (18, 95) | 134 (13, 216) | 0.132 | 68 (13, 134) | 95 (13, 171) | 0.831 |
| FSTL-1 | 4759 (3164, 9959) | 5069 (2269, 7914) | 0.404 | 3341 (968, 7741) | 5908 (3682, 12220) | 0.021 |
| Interleukin 6 | 1 (0, 4) | 5 (3, 11) | 0.005 | 2 (0, 5) | 4 (3, 8) | 0.004 |
| Interleukin 8 | 3.5 (2.9, 4.8) | 4.6 (3.2, 6.2) | 0.278 | 3.4 (2.4, 4.6) | 5.8 (3.7, 8.3) | <0.001 |
| Interleukin 15 | 0.90 (0.90, 1.30) | 1.10 (0.90, 3.00) | 0.186 | 0.92 (0.92, 2.22) | 1.22 (0.92, 2.29) | 0.452 |
| Irisin | 75 (0, 170) | 90 (0, 180) | 0.529 | 75 (0, 160) | 85 (0, 220) | 0.646 |
| Leptin | 4235 (1852, 5939) | 1637 (727, 3313) | 0.005 | 1325 (858, 2238) | 942 (491, 2150) | 0.128 |
| LIF | 2.0 (1.1, 3.9) | 2.3 (1.3, 3.5) | 0.583 | 2.7 (1.9, 3.0) | 2.9 (1.7, 3.8) | 0.271 |
| Myostatin | 146 (116, 205) | 136 (116, 1060) | 0.300 | 126 (116, 303) | 253 (108, 498) | 0.266 |
| Oncostatin M | 17 (11, 29) | 15 (9, 25) | 0.399 | 14 (7, 24) | 13 (8, 23) | 0.715 |
| Osteocrin | 13 (13, 19) | 13 (3, 24) | 0.714 | 13 (13, 18) | 13 (6, 23) | 0.258 |
| Osteonectin | 143 (112, 185) | 154 (120, 190) | 0.908 | 156 (121, 189) | 152 (117, 193) | 0.737 |
| PTHrP | 2120 (1467, 3179) | 1734 (764, 3343) | 0.306 | 1834 (907, 3892) | 2061 (1174, 3700) | 0.825 |

**Table S2.** AUC (95% confidence intervals) of ROC curves for individual myokines distinguishing between cachectic and non-cachectic patients

| **Myokine** | **AUC (95% CI)** |
| --- | --- |
| Apelin | 0.513 (0.425-0.600) |
| BDNF | 0.562 (0.499-0.626) |
| Erythropoeitin | 0.536 (0.449-0.622) |
| FABP3 | 0.597 (0.507-0.687) |
| FGF2 | 0.525 (0.436-0.614) |
| FGF21 | 0.508 (0.418-0.598) |
| Fractalkine | 0.560 (0.470-0.650) |
| FSTL-1 | 0.610 (0.518-0.701) |
| Interleukin 6 | 0.690 (0.598-0.782) |
| Interleukin 8 | 0.663 (0.581-0.746) |
| Interleukin 15 | 0.521 (0.437-0.605) |
| Irisin | 0.501 (0.431-0.571) |
| LIF | 0.506 (0.437-0.575) |
| Leptin | 0.608 (0.523-0.693) |
| Myostatin | 0.501 (0.420-0.582) |
| Oncostatin M | 0.503 (0.409-0.597) |
| Osteocrin | 0.518 (0.446-0.589) |
| Osteonectin | 0.505 (0.413-0.598) |
| PTHrP | 0.528 (0.439-0.616) |

BDNF, Brain-derived Neurotrophic Factor; FABP3, Fatty Acid-Binding Protein 3; FSTL-1, Follistatin-Like 1 Protein; FGF21, Fibroblast Growth Factor 21; FGF2, Fibroblast Growth Factor 2; LIF, Leukemia Inhibitory Factor; PTHrP, parathyroid hormone-related protein.

**Table S3.** Portal and peripheral blood levels of myokines

| **Concentration (pg/mL)** | **Peripheral blood**  **(n=24)** | **Portal blood**  **(n=24)** | **P** |
| --- | --- | --- | --- |
| Apelin | 14 (0, 29) | 0 (0, 0) | **0.003** |
| BDNF | 4161 (4161, 4161) | 4161 (4080, 4161) | 0.463 |
| Erythropoeitin | 471 (59, 730) | 122 (0, 927) | 0.213 |
| FABP3 | 2450 (1638, 4395) | 4096 (2016, 5547) | 0.398 |
| FGF2 | 40 (34, 53) | 33 (19, 50) | 0.101 |
| FGF21 | 81 (5, 369) | 22 (5, 91) | **0.028** |
| Fractalkine | 96 (84, 176) | 33 (10, 54) | **0.001** |
| FSTL-1 | 3650 (2381, 6674) | 2034 (309, 6715) | 0.246 |
| Interleukin 6 | 2 (0, 7) | 2 (0, 4) | 0.792 |
| Interleukin 8 | 6.6 (4.7, 11.2) | 5.8 (3.3, 8.5) | 0.168 |
| Interleukin 15 | 0.92 (0.92, 2.79) | 0.92 (0.92, 1.51) | 0.211 |
| Irisin | 75 (75, 172) | 75 (75, 90) | 0.520 |
| Leptin | 899 (537, 2413) | 628 (346, 1385) | **0.041** |
| LIF | 3 (3, 6) | 3 (3, 3) | 0.107 |
| Myostatin | 117 (116, 1102) | 116 (116, 311) | 0.414 |
| Oncostatin M | 17 (9, 23) | 11 (5, 15) | 0.098 |
| Osteocrin | 12.7 (12.7, 14.5) | 12.7 (12.7, 12.7) | 0.146 |
| Osteonectin | 141 (102, 176) | 109 (93, 146) | 0.093 |
| PTHrP | 1879 (1123, 3021) | 1654 (693, 1918) | 0.243 |

Data are expressed as median (interquartile range). The paired samples Wilcoxon test was used to compare groups. BDNF, Brain-Derived Neurotrophic Factor; FABP3, Fatty Acid-Binding Protein 3; FSTL-1, Follistatin-Like 1 Protein; FGF21, Fibroblast Growth Factor 21; FGF2, Fibroblast Growth Factor 2; LIF, Leukemia Inhibitory Factor; PTHrP, parathyroid hormone-related protein.

**Figure S1.** ROC curves for diagnosis of cachexia

**Figure S2.** Spearman's correlation analysis for BMI

**Figure S3.** Spearman's correlation analysis for weight loss

**Figure S4.** Serum myokine concentrations in the peripheral blood by tumour stage (n=171). Data are expressed as median (center line) and interquartile range (box). Mann–Whitney U test was used to compare median values between patients with different cancer stages.

Figure S5. Spearman correlation matrix for portal and peripheral blood levels of myokines. Only correlations with P <0.05 are coded with colour and correlation strength is indicated by colour intensity.
